# 2122. *In Vitro* Activity of Cefepime-Taniborbactam Against Clinically Significant Gram-Negative Bacteria Isolated from Patients with Cancer

**DOI:** 10.1093/ofid/ofad500.1745

**Published:** 2023-11-27

**Authors:** Bahgat Z Gerges, Y Lan Truong, Joel Rosenblatt, Ray Y Hachem, Anne-Marie Chaftari, Samuel A Shelburne, Issam I Raad

**Affiliations:** MD Anderson UT, Missouri City, Texas; UT MD Anderson Cancer Center, Houston, Texas; MD Anderson UT, Missouri City, Texas; MD Anderson UT, Missouri City, Texas; MD Anderson UT, Missouri City, Texas; MD Anderson-University of Texas, Houston,, Texas; MD Anderson UT, Missouri City, Texas

## Abstract

**Background:**

Gram negative (GN) bacterial infections are on the rise in patients with cancer (PWC). There is an urgent need for new therapies to address the rise of infections caused by multidrug resistant (MDR) GN pathogens. Taniborbactam is a β-lactamase inhibitor in combination with cefepime may offer a potential treatment option for patients with serious GN bacterial infections. This study aimed to evaluate the *in vitro* activity of a novel combination of cefepime-taniborbactam and comparators against recent Gram-negative clinical isolates from PWC.

**Methods:**

Recent GN clinical isolates from PWC were tested against cefepime-taniborbactam and comparators. Clinical and laboratory Standards Institute (CLSI) approved broth microdilution method was used. Appropriate ATCC controls were included. MIC_90_, and percent of susceptibility calculations were made using FDA breakpoints when available. We tested 100 GN isolates and further testing is ongoing.

**Results:**

Cefepime-taniborbactam and comparators antibiotics susceptibility percentage (S: %), and MIC_90_ are shown in the table below. Cefepime-taniborbactam demonstrated highly potent activity against all of tested Enterobacterales including extended spectrum Beta-lactamase (ESBL), and carbapenem-resistant Enterobacterales (CRE) as well as against *P. aeruginosa* and *Stenotrophomonas maltophilia*. At a provisional breakpoint of 16/4 mg/L, cefepime-taniborbactam showed highest activity against all tested isolates when compared to cefepime and other comparators, including activity against ESBL, CRE, MDR *P. aeruginosa* and *S. maltophilia.*

Comparative study between cefepime-taniborbactam and comparators for MIC90 (mg/L.) and Susceptibility (%) results against Gram-negative bacteria isolated from patients with cancer

**Figure:**
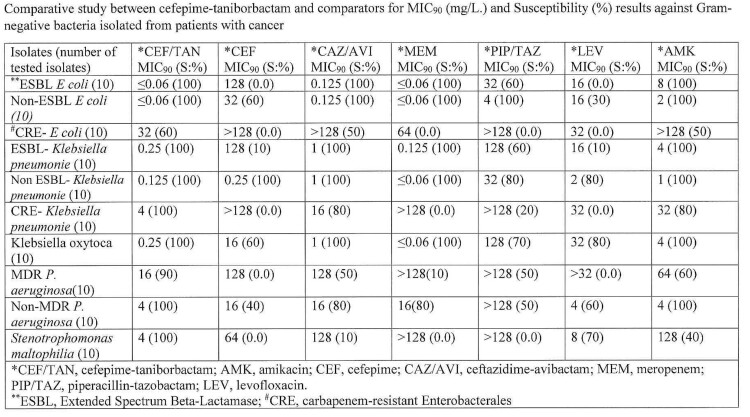

**Conclusion:**

Our data demonstrate that cefepime-taniborbactam has promising activity against clinically significant multidrug-resistant (MDR) GN bacterial pathogens isolated from PWC and it showed high activity compared to other commonly used broad spectrum antimicrobial agents.

**Disclosures:**

**Joel Rosenblatt, PhD**, Novel Anti-Infective Technologies, LLC: Licensed Technology **Issam I. Raad, Distinguished Professor**, Novel Anti-Infective Technologies, LLC: Technology License

